# Efficacy and MicroRNA-Gut Microbiota Regulatory Mechanisms of Acupuncture for Severe Chronic Constipation: Study Protocol for a Randomized Controlled Trial

**DOI:** 10.3389/fmed.2022.906403

**Published:** 2022-06-28

**Authors:** Junpeng Yao, Xiangyun Yan, Liping Chen, Yanqiu Li, Leixiao Zhang, Min Chen, Ying Li

**Affiliations:** ^1^Acupuncture and Tuina School/ the 3^nd^ Teaching Hospital, Chengdu University of Traditional Chinese Medicine, Chengdu, China; ^2^Department of Integrated Traditional and Western Medicine, West China Hospital of Sichuan University, Chengdu, China; ^3^Clinical Medicine School, Hospital of Chengdu University of Traditional Chinese Medicine, Chengdu, China

**Keywords:** severe chronic constipation, acupuncture, gut microbiota, microRNA, protocol

## Abstract

**Background:**

Severe chronic constipation (SCC) is a common functional gastrointestinal (GI) disorder associated with disruptions in GI motility. Abnormalities between gut microbiota and microRNAs (miRNAs) are implicated in the pathogenesis of GI motility in SCC. Acupuncture has been shown to improve constipation-related symptoms and rebalance the gut microbiota. This protocol proposed a plan to explore the hypothesis that the efficacy of acupuncture is associated with the crosstalk between gut microbes and miRNAs in patients with SCC.

**Methods:**

This trial is designed as a randomized, sham-controlled trial involving 80 patients and 40 healthy volunteers. A total of 80 patients with SCC (≤2 mean spontaneous, complete bowel movements per week [CSBMs]) will be randomly allocated to receive either 16-session acupuncture at true acupoints or non-penetrating sham acupuncture at non-acupoints for 4 weeks. The primary outcome will be the proportion of patients with ≥3 mean weekly CSBMs over weeks 1–4 and 5–8. Secondary efficacy endpoints include bowel movements, stool consistency, degree of straining, and the quality of life. Healthy volunteers will not receive any clinical intervention. Fasting plasma and fecal samples will be analyzed by 16S rRNA third-generation sequencing and miRNA high-throughput sequencing technologies. Finally, a tripartite network analysis will be used to investigate the interactions among clinical efficacy, miRNAs, and intestinal microbiota.

**Discussion:**

From the perspective of microRNA-gut microbiota regulatory mechanisms, our results will partially illuminate the crucial role of fecal miRNAs and intestinal microbiota to understand how acupuncture exerts its anti-constipation role.

**Trial registration:**

This trial is registered with ChiCTR2100048831, registered 18 July 2021; ethical approval has been obtained from the Sichuan Regional Ethics Review of Committee on Traditional Chinese Medicine, approval ID: 2021KL-023.

## Introduction

Chronic constipation refers to a functional bowel disorder except for irritable bowel syndrome (IBS), in which symptoms of difficult, infrequent, or incomplete defecation predominate (1). It affects ~14% of the population worldwide, with a prevalence of 4–6% in China (2, 3). Health-related quality of life (QOL) is negatively affected by the presence of chronic constipation and by its severity (4, 5). Severe chronic constipation (SCC) has distinct duration and severity of symptoms over mild and transient types, generally associated with weekly spontaneous bowel movements (SBMs) of <2 times (6). Even though empirical laxatives are the mainstay for constipated patients, a high level of dissatisfaction has been reported since they often target only 1 aspect of the disease (7). In addition, relief of symptoms is limited to the period of treatment, and efficacy tends to fade after the drug is discontinued (8). One European survey of chronic constipation revealed that almost half were using alternative treatments (acupuncture, cupping, and homeopathy), and nearly 90% of respondents expressed interest in new therapies (9).

Acupuncture is the representative of non-pharmaceutical choice with eminent advantages in the management of SCC. Our previous study revealed that the effectiveness of acupuncture was non-inferior to mosapride in increasing the weekly SBMs, but with a better safety profile (10). The effects of acupuncture could accumulate with 4-week treatments and persist throughout the 4-week follow-up. In addition to the improvement of intestinal discomforts, acupuncture can also bring better QOL to SCC patients with improved mental symptoms such as anxiety and depression (4, 11–13). Gastrointestinal (GI) motor function abnormalities are considered to be of primary importance for SCC symptoms generation. Previous basic researches demonstrated that acupuncture stimulation could improve GI motility, promote contractility of colonic tissue, and facilitate gut transit in constipated animals (14–16). However, the detailed mechanisms underlying acupuncture for SCC remain largely unknown.

Intestinal microbiota plays a critical role in GI transit. Following the onset of SCC, in addition to the decreased richness and diversity of the intestinal microbiome, microbial metabolites vary greatly (17). For example, metabolites short-chain fatty acids and γ-aminobutyric acid decrease, leading to reduced expression of the colonic serotonin transporter and intake of 5-hydroxytryptamine (5-HT), which weakens intestinal motility (18). Additionally, intestinal neuroendocrine factors may also be changed through the release of bacterial endotoxin lipopolysaccharide and bacterial final metabolites, resulting in the aggravation of constipation symptoms (19, 20). In a previous study, we have demonstrated that acupuncture could affect the production and degradation of short-chain fatty acids mainly by promoting the recovery of intestinal microbiome, thus stimulating the neural signal transduction of 5-HT signal pathway (21). Besides, acupuncture was found to be lesser effective when the intestinal microbial community was dysregulated by antibiotics (22). Nevertheless, the behind mechanism has not been clarified yet.

MicroRNA (miRNA) is a kind of endogenous, non-coding single-stranded RNA with a length of about 22 nucleotides and is highly conserved during evolution (23). It is proven that miRNA along with its related networks is involved in the imbalance of intestinal microbiota, which is the key to the pathogenesis of functional constipation (24–27). For instance, in patients with SCC, 13 miRNAs were found to be up-regulated in colonic mucosa and were identified as biomarkers for the diagnosis of disease severity (24). The miRNAs which show differential expression patterns could cause changes in intestinal microbial metabolic pathways via targeting and regulating the transcriptional levels of related genes (25, 26). In an animal experiment, related miRNA deficiency was highly likely to cause intestinal microbial disorders, while fecal miRNA transplantation could reshape the microbiota and alleviate gastrointestinal symptoms (27).

Some studies have shown that the therapeutic effect induced by acupuncture might be correlated with the regulatory mechanism of miRNAs (28–30). However, there is no report on whether the treatment of SCC by acupuncture involves the miRNA-intestinal microbiota crosstalk. Hence, a randomized, sham-controlled trial will be conducted to explore the interactions among the efficacy of acupuncture, changes of fecal miRNA, and the intestinal microbiota. In all, our study will provide initial support for the role of alternations in the microRNA-gut microbiota crosstalk during the symptom improvement induced by acupuncture.

## Methods

### Objectives

This trial aims to (1) re-evaluate the pure therapeutic effect of acupuncture for SCC; (2) identify the correlation between the gut microbiota and fecal miRNAs in patients with SCC; and (3) investigate the interactions among acupuncture-induced improvement of constipation symptoms, changes of fecal miRNAs and regulation of intestinal microbiota.

### Trial Design

This trial is designed as a randomized, sham-controlled trial with blinded participants, outcome assessor, and statistician. In this study, 80 eligible participants will be randomized to the verum acupuncture (VA) or sham acupuncture (SA) group with a ratio of 1:1. The total research period will be preceded by a 1-week run-in phase after the screening visit, then following the 4-week treatment phase, and 4-week follow-up phase. Forty healthy volunteers will also be enrolled to serve as a healthy control group. Healthy volunteers will not receive any treatment, and the correlation between gut microbiota and fecal miRNAs will be determined by comparing the constipated patients before treatment with them. The time frame of this trial is presented in Table 1.

**Table 1:** Time points of treatment assessment.

	**Enrollment**	**Baseline**	**Treatment phase**		**Follow-up phase**			
**Time point**	**Week −1**	**Week 0**	**Week 2**	**Week 4**	**Week 5**	**Week 6**	**Week7**	**Week 8**
**Screening and enrolment**
Clinical interview	**×**							
Laboratory test	**×**			**×**				
Gut microbiota analysis	**×**			**×**				
MicroRNAs analysis	**×**			**×**				
Eligibility screen	**×**							
Informed consent	**×**							
Randomization		**×**						
**Interventions**
Acupuncture			16 sessions treatment				
Sham acupuncture			16 sessions treatment				
Health control group			No acupuncture treatment				
**Assessments**
Primary outcome
Proportion of patients with ≥ 3 mean weekly CSBMs over weeks 1–4 and 5–8		**×**	**×**	**×**				**×**
**Secondary outcomes**
Proportion of patients with ≥ 1 increase of mean weekly CSBMs from baseline over weeks 1–4 and 5–8		**×**	**×**	**×**				**×**
Mean score of straining of each SBM		**×**	**×**	**×**				**×**
Mean score of stool consistency of each SBM		**×**	**×**	**×**				**×**
PAC-QOL		**×**	**×**	**×**				**×**
**Safety assessment**
Safety of acupuncture			**×**	**×**				
Adverse event			**×**	**×**				**×**
**Others**
Expectancy questionnaire		**×**						
Patients' compliance				**×**				

*CSBMs, Complete spontaneous bowel movements; SBMs, Spontaneous bowel movement; PAC-QOL, Patient Assessment of Constipation Quality of Life*.

**Figure 1:**
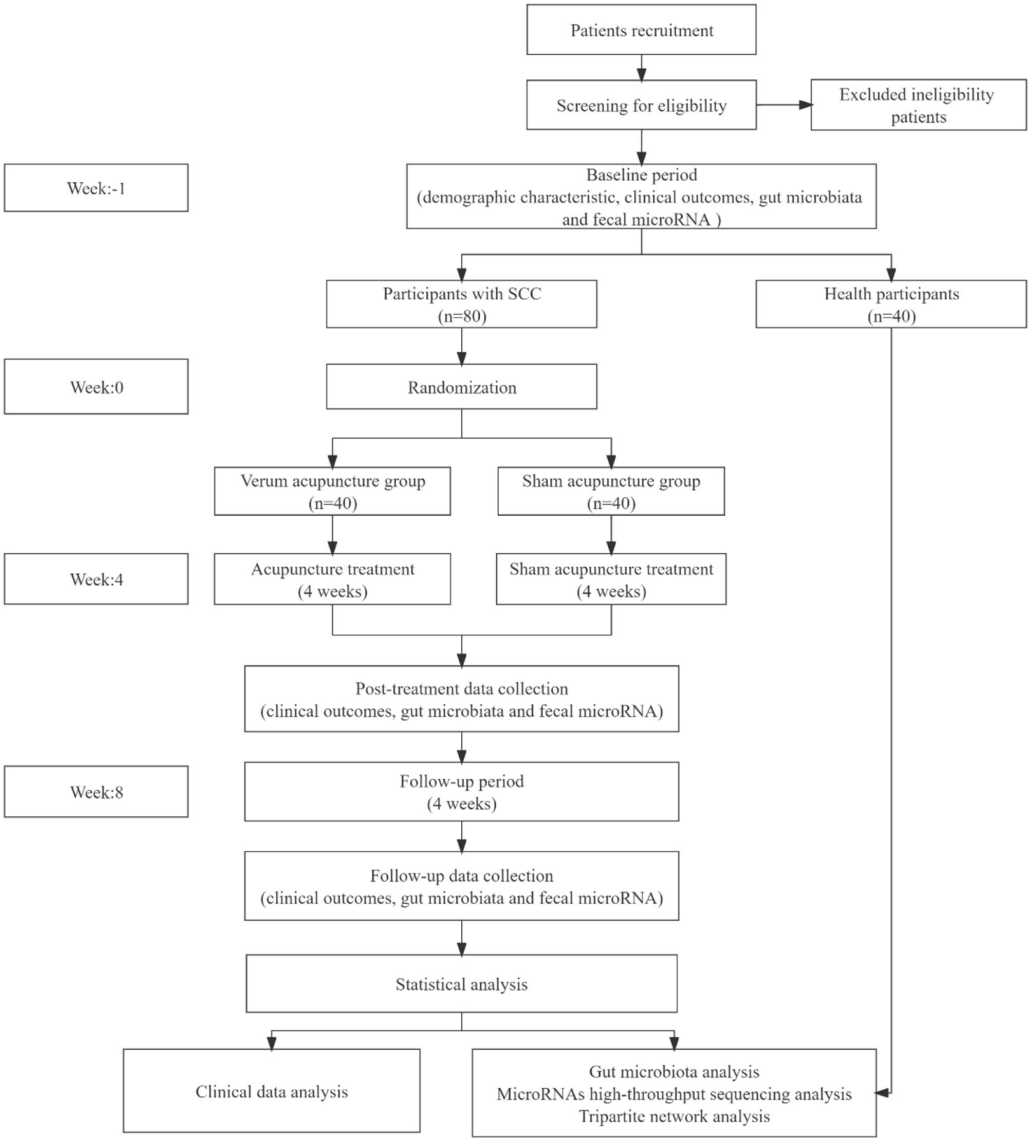
Flowchart of the trial design.

All procedures performed in this study involving human participants will in accordance with the Declaration of Helsinki (as revised in 2013). This trial has been approved by the local ethics committee (Sichuan Regional Ethics Review of Committee on Traditional Chinese Medicine, Approval ID: 2021KL-023) and was registered on the Chinese Clinical Trial Registry (ChiCTR2100048831). This protocol is reported following the Standard Protocol Items: Recommendations for Interventional Trials (SPIRIT) (31). The flow chart of this trial is shown in Figure 1.

### Participants

#### Source of Participants

This study will be conducted in the outpatient departments of First Teaching Hospital of Chengdu University of Traditional Chinese Medicine. Recruitment will be performed mainly in the following three strategies: recruitment at anorectal and gastroenterology departments, posters in local community centers, and advertisements posted on the WeChat platform.

#### Diagnostic Criteria

According to the 2016 International Functional GI Disorders-Rome IV criteria, patients must meet the following aspects (1):

1. Two or more of the following must be conformed (more than 25% of defecations): a. exertion during evacuation; b. lumpy or hard stools; c. a sense of obstruction in the anus/rectum; d. sensation of incomplete evacuation; e. manual assistance is required for defecation; f. fewer than 3 mean weekly CSBMs;
2. Loose stools rarely occur without the use of laxatives;
3. Insufficient criteria for IBS;
4. Criteria fulfilled for the last 3 months with symptom onset at least 6 months prior to diagnosis.

#### Inclusion Criteria

Participants meeting all the following conditions will be enrolled in this study:

1. Aged 18 to 60 years old;
2. History of SCC for a minimum of 6 months before the screening visit, and the activity of symptoms last for at least 3 months;
3. Two or fewer CSBMs per week, exertion in defecation, and lumpy or hard stools (Bristol Stool Form Scale [BSFS] ≤3) during the run-in period;
4. No special eating habits and not accompanied by severe anxiety, depression, or other mental disorders (SAS or SDS score <75);
5. No use of medicine for constipation during the 1 week before enrolment (except for rescue medicine), including intestinal microecological preparations, probiotics, etc.; no acupuncture treatment for constipation in the previous 1 month; no participation in any other ongoing trial;
6. Volunteered to join this trial and signed the informed consent.

#### Exclusion Criteria

Participants meeting any of the following aspects will be excluded:

1. A history of abdominal or anorectal surgery;
2. Irritable bowel syndrome and organic or drug-induced constipation; secondary to endocrine, metabolic or neurotic constipation;
3. Patients with severe cardiovascular, hepatic, or renal diseases or cognitive impairment, aphasia, or unable to cooperate with sample collection and treatment;
4. Patients with other primary diseases caused by intestinal microbial disorder (diabetes, obesity, migraine, polycystic ovary syndrome, etc.);
5. Patients during pregnancy, lactation, or with a pregnancy plan in 3 months;
6. Patients with blood coagulation dysfunction or using anticoagulants, such as Warfarin and Heparin.

### Informed Consent

When signing the informed consent, all subjects will be informed that they would be randomly assigned to VA or SA acupuncture group, with possible benefits and risks. All subjects will voluntarily sign the informed consent before participating in this study and can withdraw from the study at their discretion.

### Sample Size, Randomization and Blinding

#### Calculation of Sample Size

The formula for calculating the sample size in clinical trials is not available in the presented study. In most similar studies devoted to microRNAs and gut microbiota, the sample size is mainly 30 cases to obtain outcomes with statistical significance (24). Considering the need for further miRNA validation in an expanded cohort plus a drop-out rate of 20%, the sample size in this setting is determined to be 80 cases (40 per). Accordingly, the number of healthy volunteers will be 40 cases, with matched demographic characteristics such as age and sex.

#### Randomization

SCC participants will be divided into verum and sham acupuncture groups based on a randomization allocation card, which will be filled according to the random sequences generated by SPSS 25.0 software, including terms of random numbers, serial numbers, and allocation information, and enveloped and provided by visit order. The healthy subjects will be directly recruited as the healthy control without randomization.

#### Blinding

In this study, we will apply a type of sham acupuncture device to mask verum and sham acupuncture between patients from the two acupuncture treatment groups. The participants, other than the acupuncturists, will be single-blinded to receive treatment in separate consulting rooms, with the avoidance of communications. In the meantime, the outcomes assessors and statisticians who are responsible for statistical analysis will also be masked concerning group assignments in the process of performing this study and data analysis.

### Intervention

The Park Sham Placebo Acupuncture Device (PSD) will be used, plus a disposable sterile acupuncture needle (length 25–75 mm, diameter 0.30 mm) for the VA group and a disposable sterile retractable blunt needle with the same size for the sham acupuncture group.

All subjects will be assigned to receive acupuncture treatments for 16 times within 4 weeks, including 5 times a week on working days for the first 2 weeks, and 3 times a week (once every other day) on working day for the last 2 weeks. The operators should be acupuncturists with a certificate to practice and at least 5 years of professional qualification.

Other medications will not be allowed, except that bisacodyl (5–10 mg) and glycerin enema (110 ml) can be used for participants who fail to have a bowel movement for 3 or more consecutive days. If the patient does not respond to bisacodyl (5–10 mg) and glycerin enema, 1–2 tablets (5–10 mg) of enteric-coated bisacodyl will be permitted (11). Furthermore, the date, specific time, dose of bisacodyl or glycerin enema using will be recorded in the defecation diary and case report form (CRF).

#### Verum Acupuncture Group

**Figure 2:**
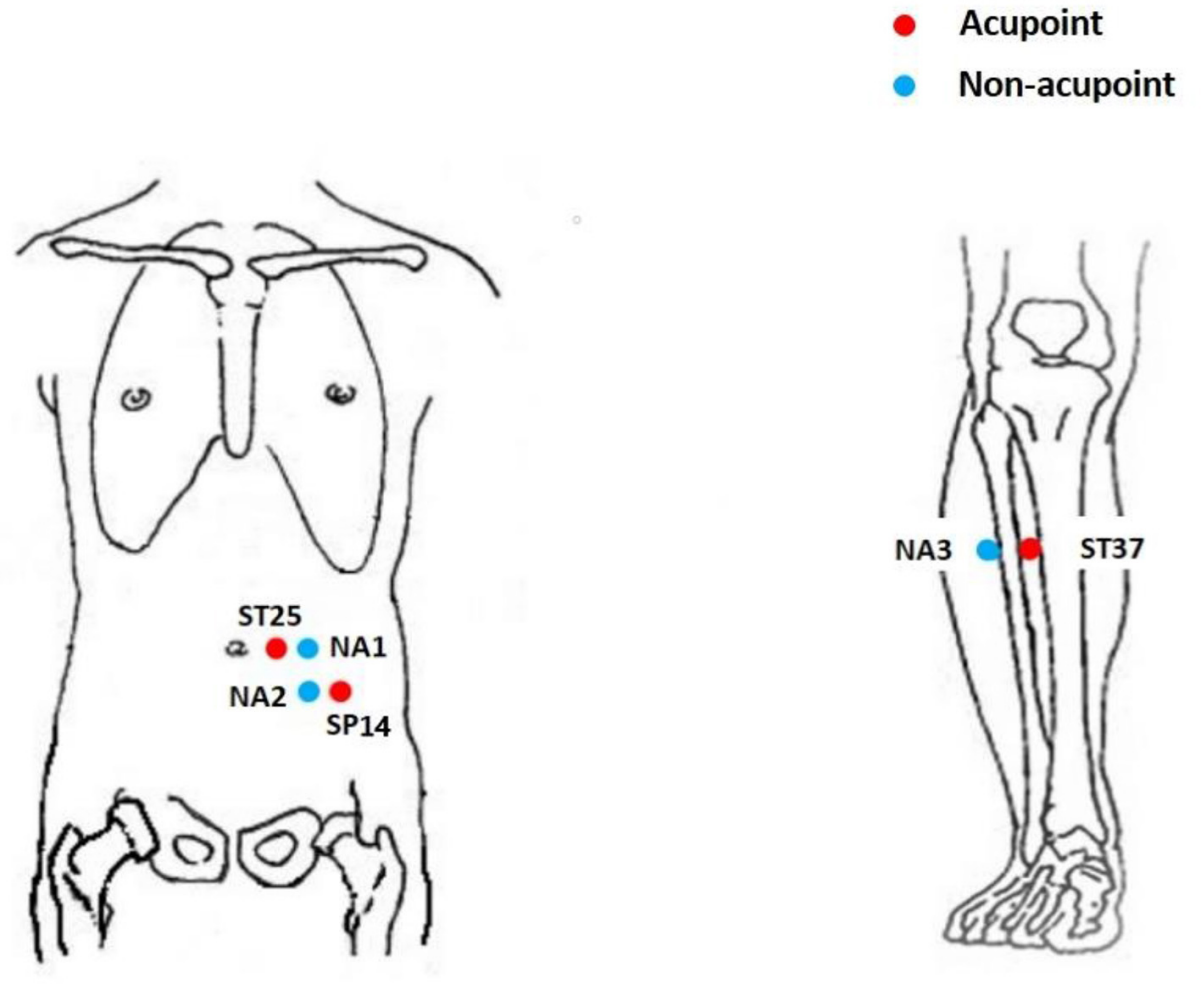
Location of acupoints and non-acupoints.

The acupoints of bilateral ST25 (*Tianshu*), SP14 (*Fujie*), and ST37 (*Shangjuxu*) will be selected for acupuncture treatment, based on previous studies and high-quality randomized controlled trials (10–12), according to the National Standard Nomenclature and Location of Acupuncture Points 2006 (GB/T12346-2006) (Table 2 and Figure 2). Moreover, BL33 (*Zhongliao*), DU20 (*Baihui*), and DU24 (*Shenting*) will be used according to the individual situation; BL33 will be used for severe straining if any, DU20 and DU24 will be used for participants accompanied with the symptoms of anxiety and depression.

**Table 2:** Acupoints and non-acupoints used in the acupuncture group.

**Acupoint**	**Location**	**Non-acupoint**	**Location**
ST25 (*Tianshu*)	On the middle portion of the abdomen, 2 cun lateral to the central of the navel (needled bilaterally)	Non-acupoint 1	On the middle of abdomen, 1 cun lateral to ST25 (*Tianshu*), the midpoint between ST25 and SP15 (*Daheng*) (needled bilaterally)
SP14 (*Fujie*)	On the lower abdomen, 1.3 cun below the central of the navel and 4 cun beside the anterior midline. (needled bilaterally)	Non-acupoint 2	On the lower abdomen, 1 cun lateral to SP14 (*Fujie*), between Stomach Meridian of Foot-Yangming and Spleen meridian of foot Taiyin (needled bilaterally)
ST37 (*Shangjuxu*)	On the outside of the lower leg, 6 cun below ST35 (*Dubi*), and on the connecting line between ST35 and ST41 (*Jiexi*). (needled bilaterally)	Non-acupoint 3	On the lateral side of the lower leg, 1 cun lateral to ST37 (*Shangjuxu*), between Stomach Meridian of Foot-Yangming and Gallbladder Meridian of Foot-Shaoyang (needled bilaterally)

The specific operations will be as follows: the rubber ring on the PSD base will be removed first to introduce a disposable filiform needle with the needle tip exposed. Then, the needles will be advanced at the selected acupoints, and the direction and depth of acupuncture will be strictly under the basic requirements of acupuncture operation. Following needle insertion, the needles will be modestly lifted and twirled to get local distension until *de qi* (a component sensation, including soreness, numbness, distension, and heaviness). Within 30 min, manual manipulation for each acupoint lasted 10 s and will be repeated three times with intervals of 10 min.

#### Sham Acupuncture Group

The sham acupoints of ST25, SP14, and ST37 will be selected to perform sham acupuncture without skin penetration (Table 2 and Figure 2). We will formulate and follow the standardized step-by-step instructions and operations to use the same rituals in the VA and SA groups as far as possible. However, instead of penetrating the skin, the blunt needles will be retracted into the hollow needle handle without penetrating the skin or reaching *de qi*. The frequency and course of treatment will be the same as those in the VA group.

### Outcome Measures

Among many persistent symptoms of SCC, reduced bowel movements are the major symptom for clinical diagnosis. An epidemiological survey showed that the most troublesome symptoms for 75% of patients are the fewer weekly bowel movements and the delayed defecation at the anus/rectum (3, 11). Therefore, bowel movements, spontaneous defecation or not, complete evacuation or not, degree of straining, stool consistency (according to Bristol stool form scale [BSFS]), and patients' QOL will be used as indicators to present the efficacy of acupuncture.

#### Primary Outcome Measurement

The primary outcome will be the proportion of patients with 3 or more mean weekly complete SBMs (CSBMs) over weeks 1–4 and 5–8. The mean weekly CSBMs will be calculated by dividing the sum of the CSBMs during weeks 1–4 or 5–8 by 4. The proportion of patients with mean weekly CSBMs ≥ 3 will be divided by the number of observed participants in this group, and then multiplied by 100% (11). One SBM refers to the bowel movement that occurs in the absence of rescue medicine, or other auxiliary techniques (laxatives, suppositories usage, or manual assistance) within the preceding 24 h; while one CSBM refers to an SBM with the sensation of complete evacuation.

#### Secondary Outcome Measurement

1. The proportion of patients with ≥ 1 increase of mean weekly CSBMs from baseline over weeks 1–4, 5–8.

The proportion of patients with mean weekly times of CSBMs ≥ 1 will be divided by the number of observed cases in this group, and then multiplied by 100%. Assessing time frame: weeks 1–4, 5–8.

2. The change from baseline in the mean score of straining of each SBM over weeks 1–4, 5–8.

The straining degree of each SBM will be evaluated using the following scale (10–12): no difficulty is 0; a little difficult, need some straining to defecate is 1 point; difficult, need straining to defecate is 2 points; very difficult, need hard straining or even auxiliary defecation is 3 points. The mean score of straining of each SBM will be calculated by dividing the total score of straining all SBMs by the sum of SBMs times in weeks 1–4 and 5–8, which will then be subtracted by the score of straining in the first visit.

3. The change from baseline in the mean score of stool consistency of each SBM over weeks 1–4, 5–8.

Participants will self-report their stool consistency of each SBM according to the 7-type BSFS (32): type 1 is separated hard lumps; type 2 is sausage-shaped but lumpy; type 3 like a sausage but with cracks on its surface; type 4 like a sausage or snake, smooth and soft; type 5 is soft blobs with clear cut edges (passed easily); type 6 is fluffy pieces with ragged edges, a mushy stool; type 7 is watery, no solid pieces, entirely liquid. The total score of stool consistency for SBMs will be divided by the summed times of SBMs during weeks 1–4 and 5–8 as the mean stool consistency for each SBMs; then will be subtracted by the score of BSFS in the first visit.

4. The change from baseline of the score of self-rated Patient Assessment of Constipation Quality of Life (PAC-QOL) at week 4.

PAC-QOL is a self-report questionnaire to evaluate the quality of life in participants with constipation, which was distributed by Mapi Research Trust in France. This questionnaire contains 28 items including 4 basic parts of physical discomfort (items 1–4), psychosocial discomfort (items 5–12), worries and concerns (items 13–23), and satisfaction (items 24–28).

The questionnaire will be scored with 5 grades, and all kinds of discomfort will be assigned 0–4 points according to the degree of discomfort from “none” to “maximum,” in which the items 18, and 25–28 are reversed. The score of each dimension is the average score of all items in this dimension, and the total score is the average score of all items.

### Collection of Fecal Samples

Fecal samples from 80 SCC patients will be collected at the onset and end of the treatment. Healthy volunteers will submit to only 1 stool collection (at baseline). Participants should be advised to maintain a normal diet (with no special changes compared with previous habits) in the run-in phase and avoid spicy, greasy, or probiotics-containing foods (such as yogurt) before stool samples are collected. Patients will receive a teaching video, as well as detailed written and oral instructions on how to collect feces using the Longsee fecal microbial genome protection solution kit (Guangdong Nanxin Medical Technology Co., Ltd.). Collection of two tubes of feces each time is required for the detection of intestinal microbiome and microRNAs. The collection location can be chosen at home or in the hospital according to the preferences of the participants. After collecting the inside of the middle part of the feces (about 3 g), it will be immediately transferred to the −80°C refrigerator in the hospital or temporarily stored in the 4°C refrigerators at home, and then quickly transferred to the researchers for microRNAs and gut microbiota analysis within 24 h.

### Safety Evaluation

Safety will be assessed by routine blood examinations, renal and liver function examinations. These indicators will be measured during the run-in period and at the end of treatment. The adverse events caused by acupuncture will be recorded, such as bleeding, pain, hematoma, syncope, local infection, and so on. Serious adverse events related to the intervention will be reported to the key researchers and recorded in the CRF. Affected participants will withdraw from the study and apply for treatment or rescue promptly.

### Statistical Analysis

#### Clinical Data Analysis

The statistical analysis plan will be determined before the initiation of the study. Demographic characteristics, baseline characteristics, and efficacy indicators will be analyzed using SPSS 25.0 software according to Full Analysis Set (FAS) and Per Protocol Set (PPS). The missing values in the FAS will be treated using the Last Observation Carried Forward method with the data closest to the first observation. The research results will be mainly based on the results of the FAS, and the results of PPS will be used as auxiliary references.

Continuous variables of normal distribution will be represented by 95% confidence interval, mean and standard deviation. Continuous variables of non-normal distribution will be described by median and inter-quartile range. Classification variables will be described in terms of percentage and frequency. The qualitative data will be compared using the chi-square test. If the quantitative data are normally distributed, we will use the analysis of variance to detect the difference between groups; otherwise, the Mann-Whitney test will be used. The data table will be recorded anonymously and statistical analysis will be carried out by independent statisticians who are blinded to the allocation.

#### Gut Microbiota Analysis

The 16S rRNA third-generation full-length sequencing technology will be used to analyze the variations in intestinal microbiota (20–22). Sequencing will be performed on the V4 region and the V3 to V4 amplification region of the 16S rRNA gene. Purified amplicons will be pooled in equimolar amounts and paired-end sequenced (2 × 250) on an Illumina MiSeq 2500 platform (Illumina, San Diego, USA) according to standard protocols. After screening, the clean sequences will be clustered into operational taxonomic units (OTUs) based on 97% identity using Uparse (version 7.0.1001). Rarefaction curves for alpha diversity will be generated to assess the efficiency of sequencing depth. Species richness and diversity will be estimated using the observed species, Chao1, Shannon index, and Simpson index. Principle coordinate analysis and partial least-squares discriminant analysis will be used to reveal differences in OTU levels between samples. The linear discriminant analysis effect size will be performed using the non-parametric factorial Kruskal–Wallis sum-rank test to assess the effect size of each differential OUT.

#### MicroRNAs High-Throughput Sequencing Analysis

High-throughput sequencing using stool samples will be performed for miRNA expression profiling to determine candidate miRNA biomarkers for the efficacy of acupuncture (24–26). Fecal miRNA levels initially will be determined using a TaqMan low-density array in pooled samples. Markedly altered miRNAs in participants will subsequently be validated using quantitative real-time polymerase chain reaction on individual samples. Agilent 2100 will be used for detection, and after qualification, these fragments will be sequenced using the Illumina NOVA platform. Bowtie software will be employed to classify and annotate sRNA, and unannotated reads will be obtained by comparing clean reads with the database. The difference between SCC patients and healthy volunteers at baseline will be analyzed to explore potential biomarkers associated with the development of SCC. Within the pool of biomarker candidates related to SCC, the difference between VA and SA at week 4 will be compared, to determine biomarkers related to the clinical efficacy.

#### Tripartite Network Analysis

The tripartite network analysis will be used to integrate information from the fecal microRNA characteristics, specific gut microbiota, and clinical data for constipated patients in the VA and SA groups. The interaction among the clinical efficacy (amelioration of SCC symptoms), microRNA characteristics (the differential expression of miRNAs in the stool), and fecal microbiome (stool microbial community) will be investigated by computing Spearman's correlations in Matlab (version R2015b). An effect size of *r* = 0.10 is considered less (1% variance explained), 0.30 moderate (9% variance explained), and 0.50 large (25% variance explained). Fisher's *r*-to-*z* transformation will be adopted to investigate the correlation coefficient difference between groups (acupuncture-sham acupuncture), using the *Z*-test. Significance will be considered at uncorrected *p* < 0.05. The Cytoscape (version 3.8.2) will be used to generate and visualize the phenome, fecal microbiome, and differentially expressed miRNAs interaction network. The networks will be presented based on selecting the gut microbiota counts and their first neighbors as nodes and all adjacent edges. The results will be shown in the line with direct (correlations with fecal microbiome) and indirect effects (clinical symptoms and differentially expressed miRNAs) with an emphasis on the association between microbiome and clinical efficacy. The comparisons between groups will be analyzed using one-way analysis of variance and independent sample *t-*test.

## Discussion

The onset of SCC is relatively complex and considered to be a result of abnormal intestinal function associated with multiple factors, such as the environment, psychology, diet, and life (1). Besides, the complexity of the pathogenesis can also be interpreted by the interactions between various pathetical mechanisms, including the changes in intestinal microbial community, weakened intestinal motility, the incidence of low-grade inflamed intestinal mucosa, and abnormal brain-intestine interactions (1, 32). With the occurrence of SCC, the quality of life and the psychological status of patients will be severely affected.

In the gene regulatory network, miRNA plays a central role different from the secondary intermediary role in the traditional central dogma, with the ability to accurately transmit and amplify signals. It has been proven that one miRNA harbors multiple target genes, and multiple miRNAs can cooperatively regulate the same target gene. In that way, there exist a series of complex regulatory networks, which are closely related to the onset and progress of various diseases with the involvement of multiple processes, such as cell proliferation, differentiation, apoptosis, and metabolism. According to the latest research, miRNAs are important targets in the research of functional constipation, owing to their functions in regulating the morphology and function of gastrointestinal motility-related cells (24–27). MiRNAs, such as miR-let-7f, miR-let-7e, and miR-200a-3p, were reported to be capable of decreasing the current density of NaV1.5 channel in colonic smooth muscle cells and thus increasing the contractile frequency and amplitude of smooth muscle strips via inhibiting the expression of target gene SCN5A (24). In an animal experiment, miR-128 was found to present significantly elevated expression in the colonic tissue of mice with functional constipation, which inhibited the p38α/M-CSF signaling pathway leading to impaired function of Cajal cells (33). The imbalance of intestinal microbiota caused by the disorders of miRNA and related regulatory networks is important for the pathogenesis of SCC. It is known that miRNAs which are differentially expressed can target a variety of intestinal bacteria, resulting in colonic motor disorder and secretion reduction (34, 35). In the meantime, the bacterial metabolites exert a negative feedback function on miRNAs, making the symptoms of constipation protracted (36).

Electroacupuncture can improve gastrointestinal peristalsis, promote the contraction of distal colon, and accelerate the whole intestinal transport in both healthy and constipated conditions (4). Acupuncture can not only regenerate damaged intestinal neurons and regulate the activities of intestinal nervous system, but also makes effects on serum motilin, ghrelin, gastrin, and bile acid (11). Based on our previous research, intestinal microbiota was identified to play an important role in the improvement of intestinal motility by acupuncture therapy in SCC patients (21). Therefore, the objective of this study is to preliminarily reveal the effector mechanism by which acupuncture regulates miRNAs and gut microbiota.

There are also certain limitations should be noted. First, it is remarkably hard to differentiate IBS-C from SCC using the ROME IV criteria, which makes the differential diagnosis more difficult. Second, the relatively small number of samples and the inability to strictly control the daily diets of participants, which may impact the findings. Third, owing to the short follow-up period, the potential value of miRNAs and gut microbiota in the long-term efficacy of acupuncture will remain unknown. In conclusion, the results of this trial are expected to not only provide clinical evidence of the efficacy of acupuncture in treating SCC, but demonstrate that this efficacy is achieved by modulating the crosstalk between fecal miRNAs and gut microbiota.

## Author Contributions

JY and YiL conceived and designed the study. JY and XY enrolled the participants and drafted the manuscript. LC and YaL conducted the treatment. LZ assessed the outcomes. MC participated in the statistical analysis. All authors revised the manuscript and approved the publication of the final version.

## Funding

This work was supported by the grant from the National Natural Science Foundation of China (No. 82074554). The funding organization had no role in the study design, conduct, or the decision-making for publication.

## Conflict of Interest

The authors declare that the research was conducted in the absence of any commercial or financial relationships that could be construed as a potential conflict of interest.

## Publisher's Note

All claims expressed in this article are solely those of the authors and do not necessarily represent those of their affiliated organizations, or those of the publisher, the editors and the reviewers. Any product that may be evaluated in this article, or claim that may be made by its manufacturer, is not guaranteed or endorsed by the publisher.

## Supplementary Material

Supplementary File 1 The SPIRIT checklist.

Supplementary File 2 Informed consent statement.

Supplementary File 3 Ethical approval file.
