# Supplementary material for: Efficacy and MicroRNA-Gut Microbiota Regulatory Mechanisms of Acupuncture for Severe Chronic Constipation: Study Protocol for a Randomized Controlled Trial
Source: Front Med (Lausanne). 2022 Jun 28;9:906403. doi: 10.3389/fmed.2022.906403 (PMC9273765; doi:10.3389/fmed.2022.906403)
Supplement: Supplementary File 2 — Informed consent statement. [file Data_Sheet_2.docx]

**INFORMED CONSENT·Informed Notice Page**

Version date and version number：20210425，V4.0

**Dear patient:**

Doctors have preliminary diagnosis you have functional constipation, we now invite you to participate in the “Study on intestinal bacteria and microRNA regulation mechanism of acupuncture in the treatment of functional constipation” topic, which is the part of the clinical research of the National Natural Science Foundation of China (general project) - “Research on intestinal bacteria remodeling and microRNA network regulation mechanism of acupuncture in the treatment of functional constipation” (Project No.: 82074554). The purpose of this study is to elaborate the mechanism of microRNA and intestinal bacteria in the treatment of functional constipation by acupuncture on the basis of the exact clinical efficacy of acupuncture in the treatment of functional constipation. This study has been approved by the Ethics Committee of the Affiliated Hospital of Chengdu University of Traditional Chinese Medicine (Ethics No.: 2021KL-023).

Before you decide whether to participate in this clinical study, please read the following as carefully as possible. It can help you understand the research and why it is necessary, the procedure and duration of the research, the important information that may benefit you after participating in the research, risks and discomfort. If you want, you can also discuss with your relatives and friends, or ask the doctor to explain and help you make a decision.

**Introduction**

**1. Research background and research purpose**

Functional constipation is a common clinical functional gastrointestinal disease in which symptoms of diffificult, infrequent, or incomplete defecation predominate. At present, digestive system diseases account for 30% of the patients in clinical internal medicine, while functional constipation accounts for about 50% of the patients in digestive department. The repeated prolongation of constipation symptoms can seriously affect the quality of life of patients, increase the risk of colorectal cancer, cardiovascular and cerebrovascular accidents, and even reduce life expectancy.

Acupuncture is effective and safe in the treatment of functional constipation. Among the 43 common diseases suitable for acupuncture and moxibustion published by WHO, constipation is specially listed. In China's investigation and Research on the dominant diseases and dominant effects of acupuncture and moxibustion in the treatment of diseases (2008), constipation and diarrhea are the top two effective diseases of acupuncture and moxibustion in the treatment of digestive system diseases. A randomized controlled clinical trial of acupuncture in the treatment of functional constipation was conducted in the national key research plan project participated by members of the research team in the early stage. The results showed that after acupuncture, the number of weekly defecation increased, the stool character and defecation difficulty were significantly improved, and the quality of life was improved. However, there is still in-depth research on the mechanism of acupuncture in the treatment of functional constipation. The purpose of this research is to scientifically explore the mechanism of microRNA and intestinal bacteria in the treatment of functional constipation.

The experiment will be conducted simultaneously at Chengdu University of traditional Chinese medicine, Affiliated Hospital of Chengdu University of traditional Chinese medicine /Sichuan Hospital of traditional Chinese medicine and National Medical Museum of Chengdu University of traditional Chinese medicine. It is expected that more than 80 subjects will participate voluntarily.

**2. Who will be invited to participate in this study?**

(1) Meet Rome IV diagnostic criteria;

(2) Aged 18 to 60 years old;

(3) History of SCC for a minimum of 6 months before the screening visit, and the activity of symptoms last for at least 3 months;

(4) Two or fewer CSBMs per week, exertion in defecation, and lumpy or hard stools (Bristol Stool Form Scale [BSFS] ≤ 3) during the run-in period;

(5) No special eating habits and not accompanied by severe anxiety, depression or other mental disorders (SAS or SDS score < 75)

(6) No use of medicine for constipation during the 1 week before enrolment (except for rescue medicine), including intestinal microecological preparations, probiotics, etc; no acupuncture treatment for constipation in the previous 1 month; no participation in any other ongoing trial;

(7) Signed informed consent and volunteered to participate in the study after knowing the treatment plan.

**3. Who will be not suitable to participate in this study**

(1) A history of abdominal or anorectal surgery;

(2) Under the age of 18 and over the age of 60;

(3) Irritable bowel syndrome and organic or drug-induced constipation; secondary to endocrine, metabolic or neurotic constipation;

(4) Patients with severe cardiovascular, hepatic or renal diseases or cognitive impairment, aphasia, or unable to cooperate with sample collection and treatment;

(5) Patients with other primary diseases caused by intestinal microbial disorder (diabetes, obesity, migraine, etc.);

(6) Patients during pregnancy, lactation, or with a pregnancy plan in three months.

(7) Patients with blood coagulation dysfunction or using anticoagulants, such as Warfarin and Heparin

**4. What will I need to do if I participate in the research?**

1. Before you are selected for the study, you will undergo the following checks to determine whether you can participate in the study:

The doctor will ask to record your medical history and conduct a physical examination and then you need to do physical and chemical tests such as blood routine and stool routine.

2. If you meet the inclusion criteria and are willing to participate in this study, the following steps will be followed:

(1) The doctor will decide which treatment you will receive according to the random number provided by the computer. The patients were divided into two groups: ① Verum acupuncture meridian group: The acupoints of bilateral ST25 (Tianshu), SP14 (Fujie) and ST37 (Shangjuxu) will be selected for the acupuncture group. ② Sham acupuncture non-meridian and non-acupoint group: Three non-meridian and non-acupoint points on both sides were be taken. You each have 1/ 2 possibilities which is divided into two in any one of the different groups. Neither you nor your doctor can know and choose the treatment in advance.

(2) In this study, the catheter is park sham placebo acupunture device (PSD) manufactured by DONGBANG Acupunture company in Korea. The specification is transparent catheter (large: φ 4 × 20mm, φ 3 × 35mm, double-sided rubber ring (φ 1 × 15mm), base (φ 4 × 15mm, φ 5 × 10mm). The filiform needle of acupuncture group is Huatuo brand disposable acupuncture needle produced by Suzhou Medical Supplies Factory Co., Ltd. the manufacturer's license is sushi YJX production license No. 2001-0020, and the registration number is sushi YJX (Zhun) Zi 2004 No. 2270202 (Specification: φ 0.25 × 25mm, φ 0.25 × 40mm). The retractable blunt needle in the sham acupuncture group was Acuprime brand disposable stainless steel sterile blunt needle imported from Britain (Specification: φ 0.25 × 25mm, φ 0.25 × 40mm).

(3) The study period will last for 9 weeks. The baseline period was 1 week, the treatment period was 4 weeks and the follow-up period was 4 weeks. The treatment course of the two groups: the first and second weeks, 5 consecutive days a week, once a day, rest for 2 days at the weekend, and then continue the treatment for the next week; In the 3rd and 4th weeks, the patients were treated 3 days a week, once every 2 days, rested for 2 days at the weekend, and then continued the treatment in the next week. The patients were treated 16 times in total. The indexes were measured at the time of enrollment, the second week, the fourth week and the eighth week after enrollment.

(4) During the treatment period, you need to keep a detailed and truthful diary of your condition (the time, times and characteristics of defecation every day). After the treatment, you need to give it to the doctor in time, and the doctor will record your symptoms and signs in detail. You need a follow-up visit at the doctor's clinic 4 weeks after the treatment. Please bring your diary to the doctor during the follow-up visit. The doctor will collect your symptoms and signs in detail each time.

(5) During the study period, you will receive a series of examinations. Your doctor will be responsible for explaining and guiding the contents and specific steps of each examination, and record your medical history and examination results on the case report form. Please accept treatment and examination in strict accordance with the doctor's instructions. At the same time, the doctor will retain your blood and stool samples before and after treatment for high-throughput sequencing detection of intestinal bacteria and microRNA. All the above examinations will be safe and will not have adverse effects on your health and condition.

3. Other matters that require your cooperation

As the subject of this study, you have some corresponding responsibilities to go to the hospital for treatment and examination on time and receive outpatient follow-up. At the same time, you are also responsible for reporting to the doctor any changes in your physical and mental aspects of the trial, whether or not you think the changes are related to the study.

You must visit the hospital according to the follow-up time agreed between the doctor and you (during the follow-up stage, the doctor may know your situation by telephone and door-to-door). It is very important because the doctor will judge whether the treatment you receive really works, and guide you to prevent and control relevant conditions in time.

It is recommended that you suspend all drugs for functional constipation during the study. If you take drugs during the study, please fill in your medication records timely and objectively, including the drugs you must continue to take if you have other complicated diseases.

**5. Possible benefits of participating in research**

You will probably benefit from this research. Such benefits include the possibility of improving your condition. The study also could help doctors and researchers to further confirm needle prick treatment of functional constipation of clinical efficacy, for the guidance of other patients with similar conditions.

The acupuncture used in this study is not the only way to treat functional constipation. You can learn about other alternative therapy from your doctor or researcher that may be available.

**6. Possible adverse reactions, risks, discomfort, and inconvenience of participating in the study**

The doctor will do his utmost to prevent and treat injuries that may be caused by this study.

You may feel acid, hemp, weight, swelling during acupunture, which are all normal reactions. Acupuncture treatment may have adverse reactions, but they are less and mild. During the course of acupuncture, some patients may have needle fainting due to your physical problems or emotional tension. It can be relieved after stopping acupuncture and taking a proper rest; Bleeding and hematoma may occur after acupuncture, which can disappear after local compression; If there is infection at the acupuncture site, your doctor will deal with it in time.

If you are treated according to the research plan, if there are adverse reactions and adverse events caused by acupuncture during the treatment process , please call the doctor at any time to call the doctor for help, and the doctor will give positive and reasonable treatment; If these adverse reactions and adverse events are confirmed The incident has caused objective damage to your body and is related to this research . This topic will give appropriate treatment measures as appropriate.

If you experience any discomfort, new changes in your condition, or any unexpected situation during the study, regardless of whether it is related to treatment, you should notify the doctor in time, and the doctor will make a judgment and give appropriate medical treatment.

During the study period, you need to go to the hospital for follow-up and do some examinations on time during the study, which may cause you trouble or inconvenience.

You can know the information and research progress related to this test at any time. If you have any problems related to this test, or you have any discomfort and injury during the test, or have questions about the rights and interests of participants in this test, you can contact Li Ying through 13708095607 (manual).

**7. Related expenses**

If you participate in this study, you will receive blood routine examination, stool routine examination and liver and kidney function examination for free, and 16 times of acupuncture treatment for free during the study period. Also, you can continue to receive 10 free follow-up acupuncture treatments according to the specific situation of your condition, so as to consolidate the curative effect after the study, .

If an adverse event occurs in a clinical trial, the medical expert committee will identify whether it is related to treatment and the research group will deal with it according to relevant regulations.

If you also combine the treatment and examination required for other diseases, it will not be included in the free range.

**8. Is personal information confidential?**

All your data for participating in this study will be kept confidential and medical recordsbe will be kept by the responsible unit and your research unit. Only the responsible unit, the research unit of clinical trials and ethics committee can directly consult your case records. Your name will not appear in any published information or report on this study.

We will make every effort to protect the privacy of your personal medical information to the extent permitted by law.

**9. How to get more information?**

You can ask any questions about this study at any time and get answers accordingly.

If there is any important new information during the study that may affect your willingness to continue to participate in the study, your doctor will inform you in time.

**10. Can voluntarily choose to participate in the study and withdraw from the study**

Whether to participate in the study depends entirely on your wishes. You may refuse to participate in the study or withdraw from the study at any time during the study, which will not affect the relationship between you and the doctor, and will not affect the loss of your medical or other interests.

For your best interests, the doctor or investigator may suspend your participation in the study at any time during the study.

If you withdraw from the study for any reason, you may be asked about your use of acupuncture. If the doctor thinks it is necessary, you may also be required to undergo laboratory and physical examinations.

**11. What should I do now?**

Whether or not to participate in this study is up to you (and your family).

Before you make a decision to participate in the study, please ask your doctor if you can.

Thank you for reading the above materials. If you decide to participate in this study, please tell your doctor, he or she will arrange all matters related to the study for you.

Please keep this information.

**INFORMED CONSENT FORM, CONSENT SIGNATURE PAGE**

**Clinical Research Project：** Study on intestinal bacteria and microRNA regulation mechanism of acupuncture for functional constipation

**Project undertaking unit：** Chengdu University of Traditional Chinese Medicine

**Consent statement:**

1, I have read the above introduction to this study and have the opportunity to discuss and ask questions with doctors about this study. All the questions I have raised were answered satisfactorily.

2, I know the possible risks and benefits of participating in this study. I know that participation in the study is voluntary and I confirm that I have sufficient time to consider this and understand:

(1) I can consult the doctor for more information at any time.

(2) I can withdraw from this study at any time without discrimination or retaliation, and medical treatment and rights will not be affected.

(3) I also know that if I withdraw from the study halfway, if I tell the doctor about the change of my condition and complete the corresponding physical and chemical examination, it will be very beneficial to the whole study.

(4) If I need to take any other medication due to the change of my condition, I will seek the advice of the doctor in advance, or tell the doctor truthfully afterwards.

(5) I agree that applicant, the research unit of the clinical trial and the ethics committee departments can consult my research materials.

(6) I will get a signed and dated copy of the informed consent.

In the end, I decided to agree to participate in this study, and promised to follow the doctor's advice as much as possible.

Patients Signature: _ _ _ _ Day _ _ _ _ Month _ _ Year

Contact mobile phone:

I confirm that I have explained to the patient the details of the trial, including its rights and possible benefits and risks, and gave it a copy of the signed informed consent.

Doctor Signature:                 　    _ _ _ _ Day _ _ _ _ Month _ _ Year

Contact mobile phone:
