# Supplementary material for: Efficacy and MicroRNA-Gut Microbiota Regulatory Mechanisms of Acupuncture for Severe Chronic Constipation: Study Protocol for a Randomized Controlled Trial
Source: Front Med (Lausanne). 2022 Jun 28;9:906403. doi: 10.3389/fmed.2022.906403 (PMC9273765; doi:10.3389/fmed.2022.906403)
Supplement: Supplementary File 3 — Ethical approval file. [file Data_Sheet_3.docx]

Sichuan Regional Ethics Review of Committee on Traditional Chinese Medicine

Medical Ethic Committee of Affiliated Hospital of Chengdu University of Traditional

Chinese Medicine

Ethical Review Comments

| Approval number | 2021KL-023 | | |
| --- | --- | --- | --- |
| Project title | Efficacy and MicroRNA-Gut Microbiota Regulatory Mechanisms of Acupuncture for Severe Chronic Constipation | | |
| Funding | The National Natural Science Foundation of China “Research on intestinal flora remodeling and microRNA network regulation mechanism of acupuncture treatment for functional constipation | | |
| Clinical research institution | Chengdu University of Traditional Chinese Medicine | | |
| Principle investigator | Li Ying | | |
| Type | Review | Method | Rapid review |
| Date | 27^th^ April 2021 | Location | Office of Ethics Committee |
| Committee member | Zhou Jianwei | | |
| Files for review | 1. Application table for reexamination 2. Revised informed consent(Version date and No: 20210425) 3. Study medical record and/ or case report form, subject diary and other questionnaire( 202210425, V4.0) | | |
| Review comments |  | | |
| (A brief translation of this part) After review by the ethics committee, it is agreed that the research protocol shall be performed in accordance with relevant guidelines and regulations. Researchers should carry out clinical research as soon as possible in accordance with the GCP principles and the reviewed protocol. In the research process, suggestions and suggestions are put forward from the following aspects. | | | |
| Period of validity | 27^th^ April 2021-26^th^ April 2024 | Tracking period | 12 months |
| Contact | Ma Xitao: 02887783139, [ethicscd@126.com](mailto:ethicscd@126.com) | | |
| Signature of Chief committee | He Chengshi | | |
| Sichuan Regional Ethics Review of Committee on Traditional Chinese Medicine  Medical Ethic Committee of Affiliated Hospital of Chengdu University of Traditional Chinese Medicine | | | |
| Date: 27^th^ April 2021 | | | |
